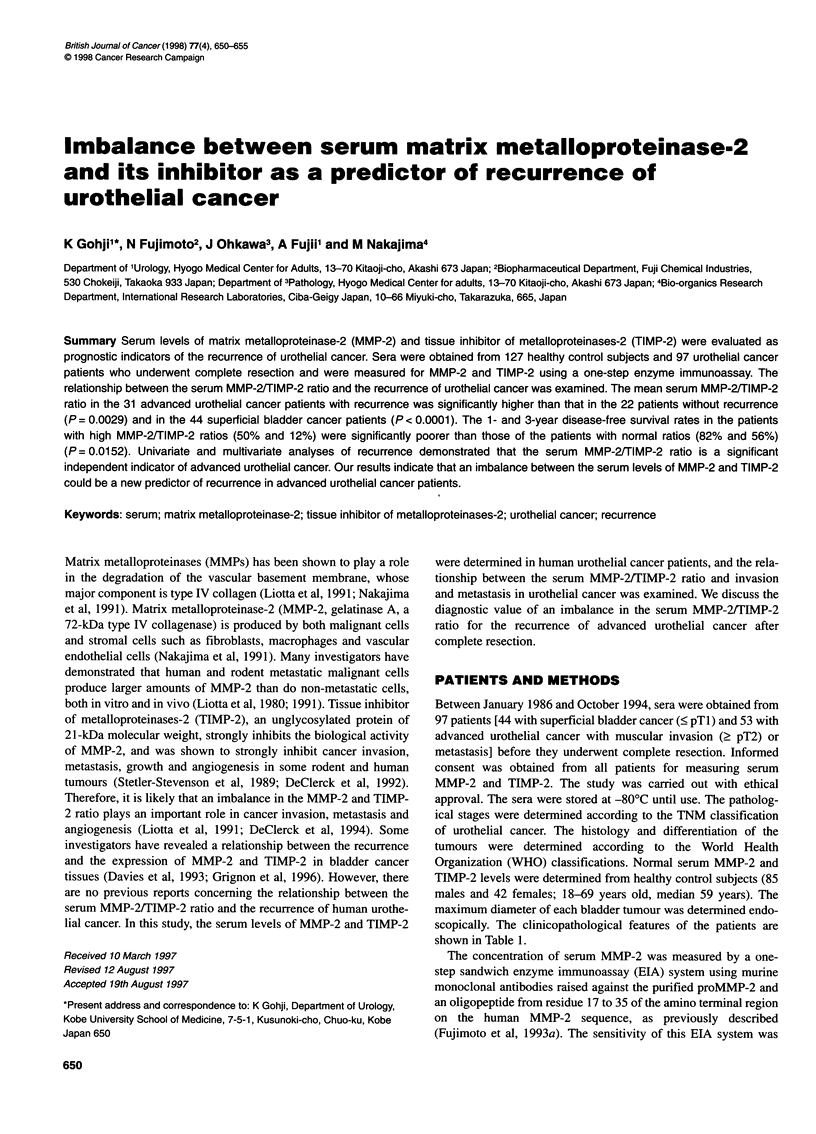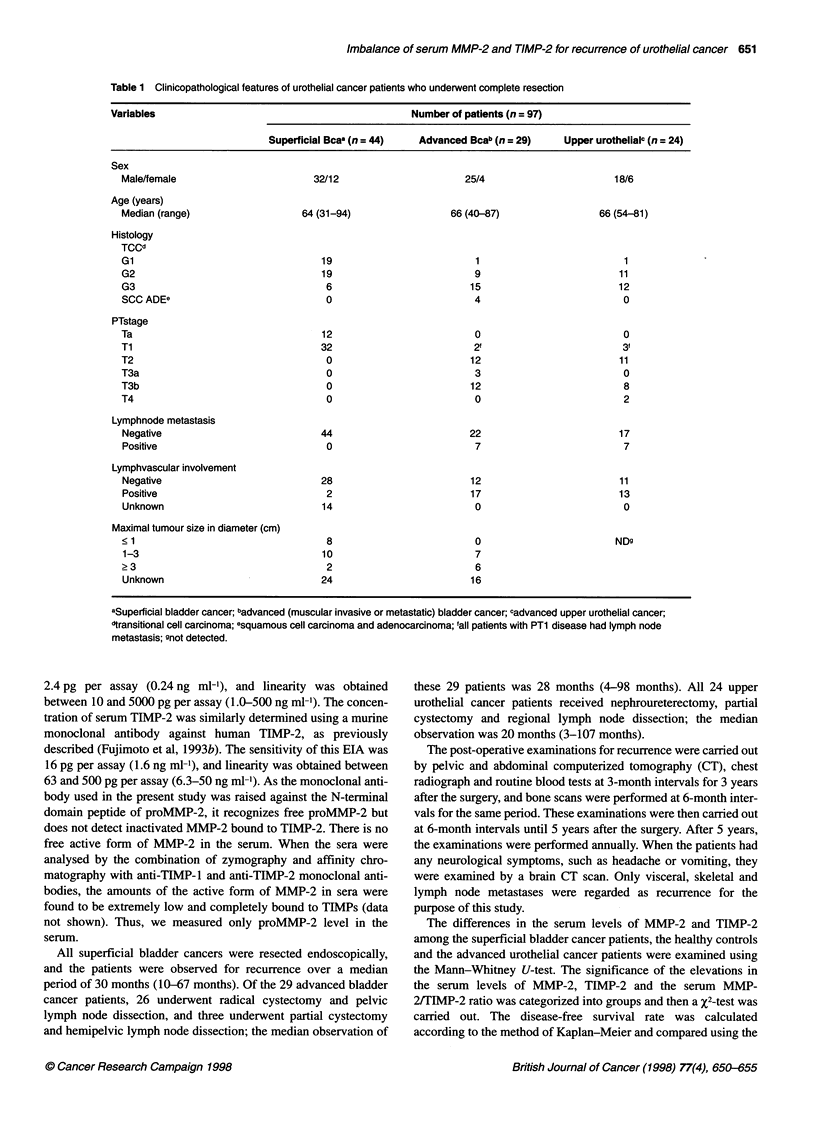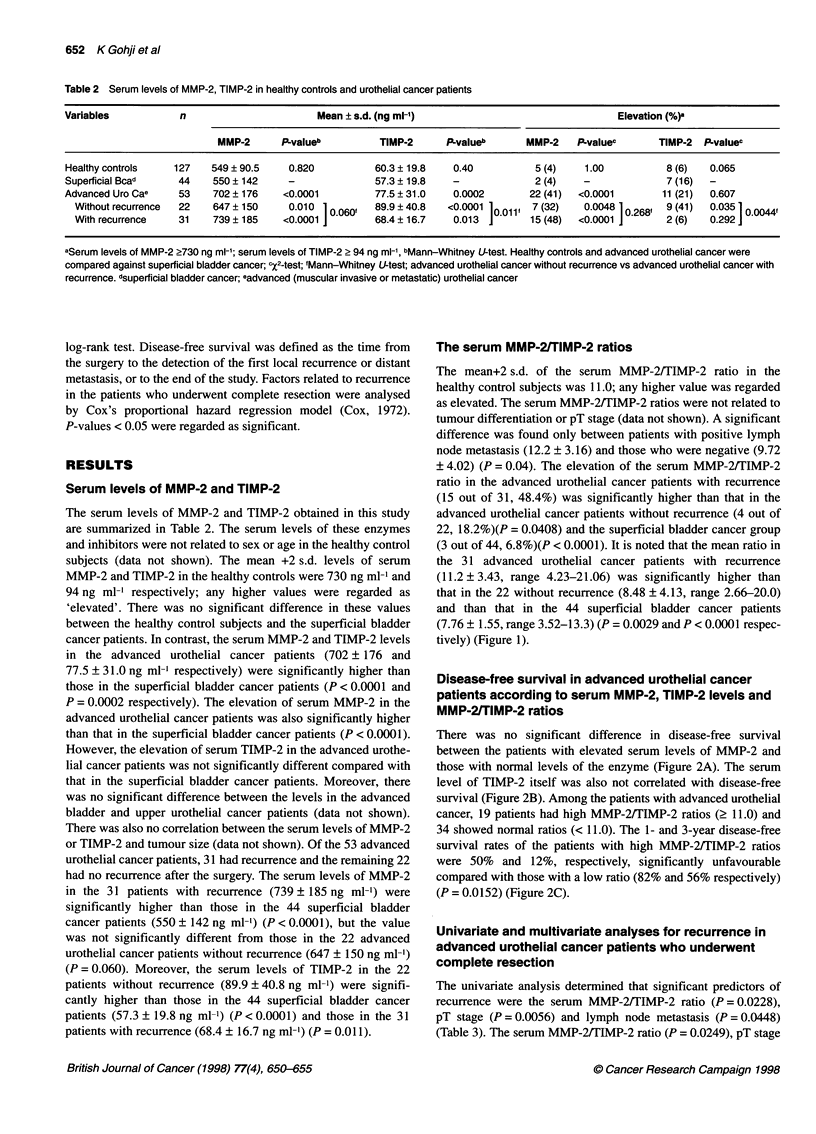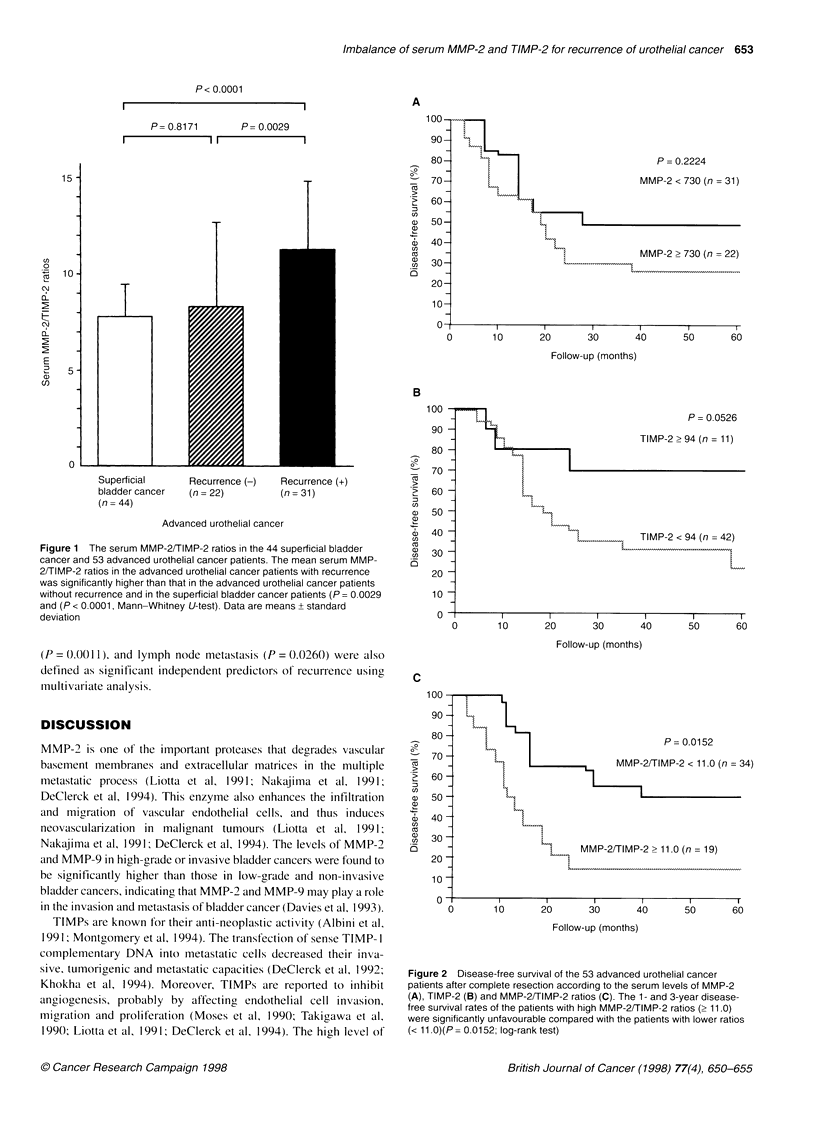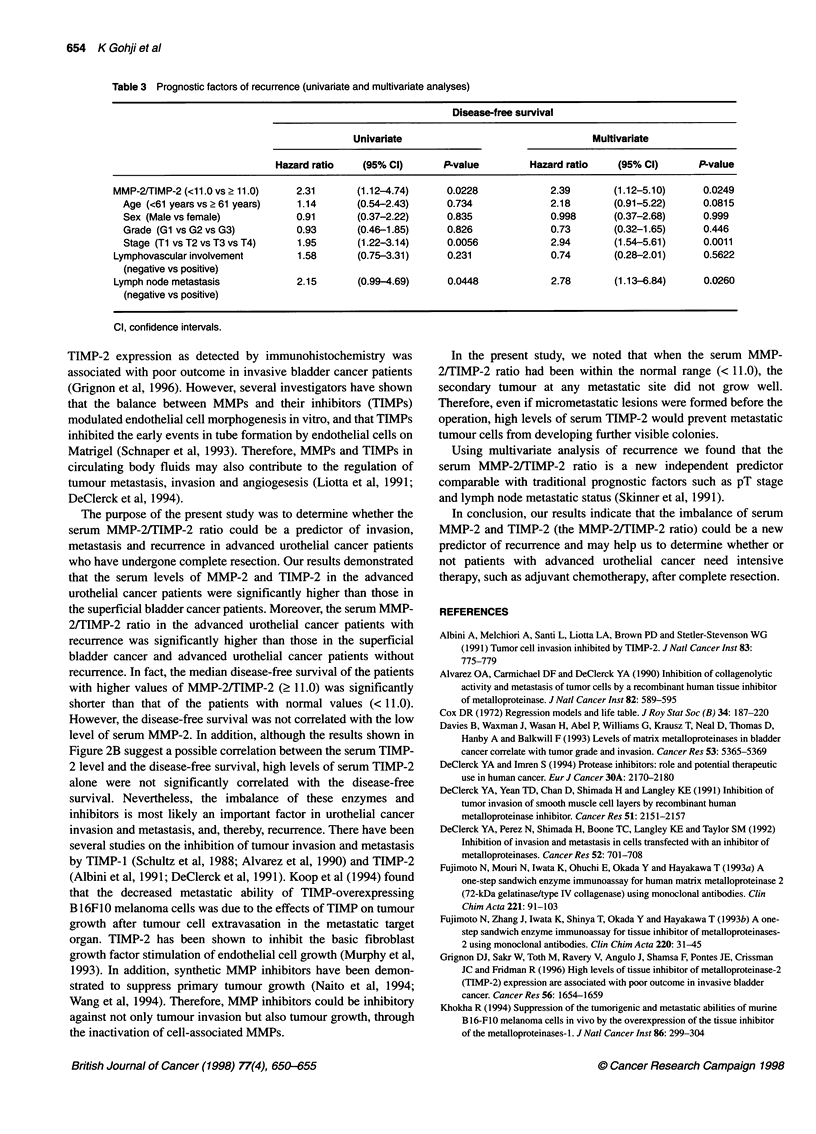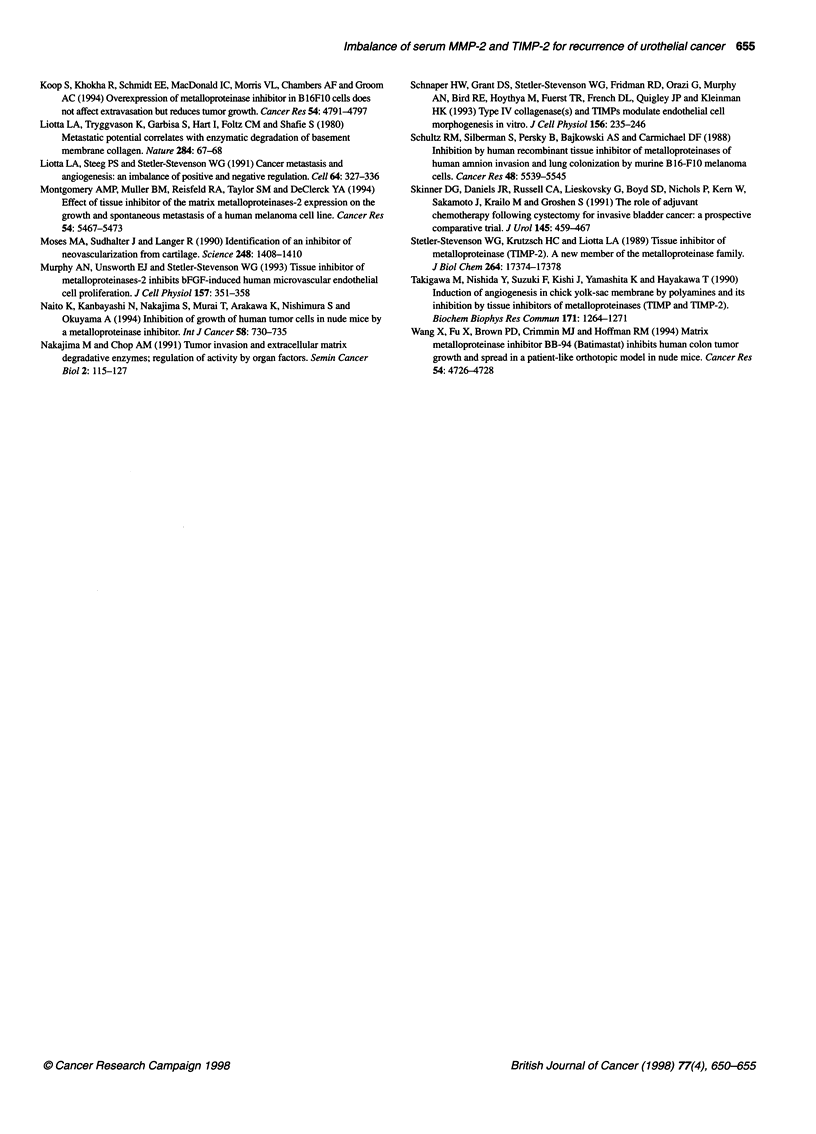# Imbalance between serum matrix metalloproteinase-2 and its inhibitor as a predictor of recurrence of urothelial cancer.

**DOI:** 10.1038/bjc.1998.104

**Published:** 1998-02

**Authors:** K. Gohji, N. Fujimoto, J. Ohkawa, A. Fujii, M. Nakajima

**Affiliations:** Department of Urology, Hyogo Medical Center for Adults, Akashi, Japan.

## Abstract

Serum levels of matrix metalloproteinase-2 (MMP-2) and tissue inhibitor of metalloproteinases-2 (TIMP-2) were evaluated as prognostic indicators of the recurrence of urothelial cancer. Sera were obtained from 127 healthy control subjects and 97 urothelial cancer patients who underwent complete resection and were measured for MMP-2 and TIMP-2 using a one-step enzyme immunoassay. The relationship between the serum MMP-2/TIMP-2 ratio and the recurrence of urothelial cancer was examined. The mean serum MMP-2/TIMP-2 ratio in the 31 advanced urothelial cancer patients with recurrence was significantly higher than that in the 22 patients without recurrence (P = 0.0029) and in the 44 superficial bladder cancer patients (P < 0.0001). The 1- and 3-year disease-free survival rates in the patients with high MMP-2/TIMP-2 ratios (50% and 12%) were significantly poorer than those of the patients with normal ratios (82% and 56%) (P = 0.0152). Univariate and multivariate analyses of recurrence demonstrated that the serum MMP-2/TIMP-2 ratio is a significant independent indicator of advanced urothelial cancer. Our results indicate that an imbalance between the serum levels of MMP-2 and TIMP-2 could be a new predictor of recurrence in advanced urothelial cancer patients.


British Journal of Cancer (1998) 77(4), 650–655
© 1998 Cancer Research Campaign

### Imbalance between serum matrix metalloproteinase-2 and its inhibitor as a predictor of recurrence of urothelial cancer

**K Gohji[1]*, N Fujimoto[2], J Ohkawa[3], A Fujii[1] and M Nakajima[4]**

Department of [1]Urology, Hyogo Medical Center for Adults, 13–70 Kitaoji-cho, Akashi 673 Japan; [2]Biopharmaceutical Department, Fuji Chemical Industries, 530 Chokeiji, Takaoka 933 Japan; Department of [3]Pathology, Hyogo Medical Center for adults, 13–70 Kitaoji-cho, Akashi 673 Japan; [4]Bio-organics Research Department, International Research Laboratories, Ciba-Geigy Japan, 10–66 Miyuki-cho, Takarazuka, 665, Japan

**Summary** Serum levels of matrix metalloproteinase-2 (MMP-2) and tissue inhibitor of metalloproteinases-2 (TIMP-2) were evaluated as prognostic indicators of the recurrence of urothelial cancer. Sera were obtained from 127 healthy control subjects and 97 urothelial cancer patients who underwent complete resection and were measured for MMP-2 and TIMP-2 using a one-step enzyme immunoassay. The relationship between the serum MMP-2/TIMP-2 ratio and the recurrence of urothelial cancer was examined. The mean serum MMP-2/TIMP-2 ratio in the 31 advanced urothelial cancer patients with recurrence was significantly higher than that in the 22 patients without recurrence ($P = 0.0029$) and in the 44 superficial bladder cancer patients ($P < 0.0001$). The 1- and 3-year disease-free survival rates in the patients with high MMP-2/TIMP-2 ratios (50% and 12%) were significantly poorer than those of the patients with normal ratios (82% and 56%) ($P = 0.0152$). Univariate and multivariate analyses of recurrence demonstrated that the serum MMP-2/TIMP-2 ratio is a significant independent indicator of advanced urothelial cancer. Our results indicate that an imbalance between the serum levels of MMP-2 and TIMP-2 could be a new predictor of recurrence in advanced urothelial cancer patients.

**Keywords**: serum; matrix metalloproteinase-2; tissue inhibitor of metalloproteinases-2; urothelial cancer; recurrence

Matrix metalloproteinases (MMPs) has been shown to play a role in the degradation of the vascular basement membrane, whose major component is type IV collagen (Liotta et al, 1991; Nakajima et al, 1991). Matrix metalloproteinase-2 (MMP-2, gelatinase A, a 72-kDa type IV collagenase) is produced by both malignant cells and stromal cells such as fibroblasts, macrophages and vascular endothelial cells (Nakajima et al, 1991). Many investigators have demonstrated that human and rodent metastatic malignant cells produce larger amounts of MMP-2 than do non-metastatic cells, both in vitro and in vivo (Liotta et al, 1980; 1991). Tissue inhibitor of metalloproteinases-2 (TIMP-2), an unglycosylated protein of 21-kDa molecular weight, strongly inhibits the biological activity of MMP-2, and was shown to strongly inhibit cancer invasion, metastasis, growth and angiogenesis in some rodent and human tumours (Stetler-Stevenson et al, 1989; DeClerck et al, 1992). Therefore, it is likely that an imbalance in the MMP-2 and TIMP-2 ratio plays an important role in cancer invasion, metastasis and angiogenesis (Liotta et al, 1991; DeClerck et al, 1994). Some investigators have revealed a relationship between the recurrence and the expression of MMP-2 and TIMP-2 in bladder cancer tissues (Davies et al, 1993; Grignon et al, 1996). However, there are no previous reports concerning the relationship between the serum MMP-2/TIMP-2 ratio and the recurrence of human urothelial cancer. In this study, the serum levels of MMP-2 and TIMP-2 were determined in human urothelial cancer patients, and the relationship between the serum MMP-2/TIMP-2 ratio and invasion and metastasis in urothelial cancer was examined. We discuss the diagnostic value of an imbalance in the serum MMP-2/TIMP-2 ratio for the recurrence of advanced urothelial cancer after complete resection.

*Received 10 March 1997*
*Revised 12 August 1997*
*Accepted 19th August 1997*

*Present address and correspondence to: K Gohji, Department of Urology, Kobe University School of Medicine, 7-5-1, Kusunoki-cho, Chuo-ku, Kobe Japan 650

### PATIENTS AND METHODS

Between January 1986 and October 1994, sera were obtained from 97 patients [44 with superficial bladder cancer (≤ pT1) and 53 with advanced urothelial cancer with muscular invasion (≥ pT2) or metastasis] before they underwent complete resection. Informed consent was obtained from all patients for measuring serum MMP-2 and TIMP-2. The study was carried out with ethical approval. The sera were stored at –80°C until use. The pathological stages were determined according to the TNM classification of urothelial cancer. The histology and differentiation of the tumours were determined according to the World Health Organization (WHO) classifications. Normal serum MMP-2 and TIMP-2 levels were determined from healthy control subjects (85 males and 42 females; 18–69 years old, median 59 years). The maximum diameter of each bladder tumour was determined endoscopically. The clinicopathological features of the patients are shown in Table 1.

The concentration of serum MMP-2 was measured by a one-step sandwich enzyme immunoassay (EIA) system using murine monoclonal antibodies raised against the purified proMMP-2 and an oligopeptide from residue 17 to 35 of the amino terminal region on the human MMP-2 sequence, as previously described (Fujimoto et al, 1993*a*). The sensitivity of this EIA system was

**Table 1** Clinicopathological features of urothelial cancer patients who underwent complete resection

| Variables | Number of patients (*n* = 97) | | |
|---|---|---|---|
| | Superficial Bca[a] (*n* = 44) | Advanced Bca[b] (*n* = 29) | Upper urothelial[c] (*n* = 24) |
| Sex | | | |
| Male/female | 32/12 | 25/4 | 18/6 |
| Age (years) | | | |
| Median (range) | 64 (31–94) | 66 (40–87) | 66 (54–81) |
| Histology | | | |
| TCC[d] | | | |
| G1 | 19 | 1 | 1 |
| G2 | 19 | 9 | 11 |
| G3 | 6 | 15 | 12 |
| SCC ADE[e] | 0 | 4 | 0 |
| PTstage | | | |
| Ta | 12 | 0 | 0 |
| T1 | 32 | 2[f] | 3[f] |
| T2 | 0 | 12 | 11 |
| T3a | 0 | 3 | 0 |
| T3b | 0 | 12 | 8 |
| T4 | 0 | 0 | 2 |
| Lymphnode metastasis | | | |
| Negative | 44 | 22 | 17 |
| Positive | 0 | 7 | 7 |
| Lymphvascular involvement | | | |
| Negative | 28 | 12 | 11 |
| Positive | 2 | 17 | 13 |
| Unknown | 14 | 0 | 0 |
| Maximal tumour size in diameter (cm) | | | |
| ≤ 1 | 8 | 0 | ND[g] |
| 1–3 | 10 | 7 | |
| ≥ 3 | 2 | 6 | |
| Unknown | 24 | 16 | |

[a]Superficial bladder cancer; [b]advanced (muscular invasive or metastatic) bladder cancer; [c]advanced upper urothelial cancer; [d]transitional cell carcinoma; [e]squamous cell carcinoma and adenocarcinoma; [f]all patients with PT1 disease had lymph node metastasis; [g]not detected.

2.4 pg per assay (0.24 ng $ml^{-1}$), and linearity was obtained between 10 and 5000 pg per assay (1.0–500 ng $ml^{-1}$). The concentration of serum TIMP-2 was similarly determined using a murine monoclonal antibody against human TIMP-2, as previously described (Fujimoto et al, 1993*b*). The sensitivity of this EIA was 16 pg per assay (1.6 ng $ml^{-1}$), and linearity was obtained between 63 and 500 pg per assay (6.3–50 ng $ml^{-1}$). As the monoclonal antibody used in the present study was raised against the N-terminal domain peptide of proMMP-2, it recognizes free proMMP-2 but does not detect inactivated MMP-2 bound to TIMP-2. There is no free active form of MMP-2 in the serum. When the sera were analysed by the combination of zymography and affinity chromatography with anti-TIMP-1 and anti-TIMP-2 monoclonal antibodies, the amounts of the active form of MMP-2 in sera were found to be extremely low and completely bound to TIMPs (data not shown). Thus, we measured only proMMP-2 level in the serum.

All superficial bladder cancers were resected endoscopically, and the patients were observed for recurrence over a median period of 30 months (10–67 months). Of the 29 advanced bladder cancer patients, 26 underwent radical cystectomy and pelvic lymph node dissection, and three underwent partial cystectomy and hemipelvic lymph node dissection; the median observation of these 29 patients was 28 months (4–98 months). All 24 upper urothelial cancer patients received nephroureterectomy, partial cystectomy and regional lymph node dissection; the median observation was 20 months (3–107 months).

The post-operative examinations for recurrence were carried out by pelvic and abdominal computerized tomography (CT), chest radiograph and routine blood tests at 3-month intervals for 3 years after the surgery, and bone scans were performed at 6-month intervals for the same period. These examinations were then carried out at 6-month intervals until 5 years after the surgery. After 5 years, the examinations were performed annually. When the patients had any neurological symptoms, such as headache or vomiting, they were examined by a brain CT scan. Only visceral, skeletal and lymph node metastases were regarded as recurrence for the purpose of this study.

The differences in the serum levels of MMP-2 and TIMP-2 among the superficial bladder cancer patients, the healthy controls and the advanced urothelial cancer patients were examined using the Mann–Whitney *U*-test. The significance of the elevations in the serum levels of MMP-2, TIMP-2 and the serum MMP-2/TIMP-2 ratio was categorized into groups and then a $\chi^2$-test was carried out. The disease-free survival rate was calculated according to the method of Kaplan–Meier and compared using the

**Table 2** Serum levels of MMP-2, TIMP-2 in healthy controls and urothelial cancer patients

| Variables | *n* | Mean ± s.d. (ng ml$^{-1}$) | | | | | | Elevation (%)[a] | | | | | |
|---|---|---|---|---|---|---|---|---|---|---|---|---|---|
| | | MMP-2 | *P*-value[b] | | TIMP-2 | *P*-value[b] | | MMP-2 | *P*-value[c] | | TIMP-2 | *P*-value[c] | |
| Healthy controls | 127 | 549 ± 90.5 | 0.820 | | 60.3 ± 19.8 | 0.40 | | 5 (4) | 1.00 | | 8 (6) | 0.065 | |
| Superficial Bca[d] | 44 | 550 ± 142 | – | | 57.3 ± 19.8 | – | | 2 (4) | – | | 7 (16) | – | |
| Advanced Uro Ca[e] | 53 | 702 ± 176 | <0.0001 | | 77.5 ± 31.0 | 0.0002 | | 22 (41) | <0.0001 | | 11 (21) | 0.607 | |
| Without recurrence | 22 | 647 ± 150 | 0.010 | 0.060[f] | 89.9 ± 40.8 | <0.0001 | 0.011[f] | 7 (32) | 0.0048 | 0.268[f] | 9 (41) | 0.035 | 0.0044[f] |
| With recurrence | 31 | 739 ± 185 | <0.0001 | | 68.4 ± 16.7 | 0.013 | | 15 (48) | <0.0001 | | 2 (6) | 0.292 | |

[a]Serum levels of MMP-2 ≥730 ng ml$^{-1}$; serum levels of TIMP-2 ≥ 94 ng ml$^{-1}$, [b]Mann–Whitney *U*-test. Healthy controls and advanced urothelial cancer were compared against superficial bladder cancer; [c]$\chi^2$-test; [f]Mann–Whitney *U*-test; advanced urothelial cancer without recurrence vs advanced urothelial cancer with recurrence. [d]superficial bladder cancer; [e]advanced (muscular invasive or metastatic) urothelial cancer log-rank test. Disease-free survival was defined as the time from the surgery to the detection of the first local recurrence or distant metastasis, or to the end of the study. Factors related to recurrence in the patients who underwent complete resection were analysed by Cox's proportional hazard regression model (Cox, 1972). *P*-values < 0.05 were regarded as significant.

### RESULTS

### Serum levels of MMP-2 and TIMP-2

The serum levels of MMP-2 and TIMP-2 obtained in this study are summarized in Table 2. The serum levels of these enzymes and inhibitors were not related to sex or age in the healthy control subjects (data not shown). The mean +2 s.d. levels of serum MMP-2 and TIMP-2 in the healthy controls were 730 ng ml$^{-1}$ and 94 ng ml$^{-1}$ respectively; any higher values were regarded as 'elevated'. There was no significant difference in these values between the healthy control subjects and the superficial bladder cancer patients. In contrast, the serum MMP-2 and TIMP-2 levels in the advanced urothelial cancer patients (702 ± 176 and 77.5 ± 31.0 ng ml$^{-1}$ respectively) were significantly higher than those in the superficial bladder cancer patients ($P < 0.0001$ and $P = 0.0002$ respectively). The elevation of serum MMP-2 in the advanced urothelial cancer patients was also significantly higher than that in the superficial bladder cancer patients ($P < 0.0001$). However, the elevation of serum TIMP-2 in the advanced urothelial cancer patients was not significantly different compared with that in the superficial bladder cancer patients. Moreover, there was no significant difference between the levels in the advanced bladder and upper urothelial cancer patients (data not shown). There was also no correlation between the serum levels of MMP-2 or TIMP-2 and tumour size (data not shown). Of the 53 advanced urothelial cancer patients, 31 had recurrence and the remaining 22 had no recurrence after the surgery. The serum levels of MMP-2 in the 31 patients with recurrence (739 ± 185 ng ml$^{-1}$) were significantly higher than those in the 44 superficial bladder cancer patients (550 ± 142 ng ml$^{-1}$) ($P < 0.0001$), but the value was not significantly different from those in the 22 advanced urothelial cancer patients without recurrence (647 ± 150 ng ml$^{-1}$) ($P = 0.060$). Moreover, the serum levels of TIMP-2 in the 22 patients without recurrence (89.9 ± 40.8 ng ml$^{-1}$) were significantly higher than those in the 44 superficial bladder cancer patients (57.3 ± 19.8 ng ml$^{-1}$) ($P < 0.0001$) and those in the 31 patients with recurrence (68.4 ± 16.7 ng ml$^{-1}$) ($P = 0.011$).

### The serum MMP-2/TIMP-2 ratios

The mean+2 s.d. of the serum MMP-2/TIMP-2 ratio in the healthy control subjects was 11.0; any higher value was regarded as elevated. The serum MMP-2/TIMP-2 ratios were not related to tumour differentiation or pT stage (data not shown). A significant difference was found only between patients with positive lymph node metastasis (12.2 ± 3.16) and those who were negative (9.72 ± 4.02) ($P = 0.04$). The elevation of the serum MMP-2/TIMP-2 ratio in the advanced urothelial cancer patients with recurrence (15 out of 31, 48.4%) was significantly higher than that in the advanced urothelial cancer patients without recurrence (4 out of 22, 18.2%)($P = 0.0408$) and the superficial bladder cancer group (3 out of 44, 6.8%)($P < 0.0001$). It is noted that the mean ratio in the 31 advanced urothelial cancer patients with recurrence (11.2 ± 3.43, range 4.23–21.06) was significantly higher than that in the 22 without recurrence (8.48 ± 4.13, range 2.66–20.0) and than that in the 44 superficial bladder cancer patients (7.76 ± 1.55, range 3.52–13.3) ($P = 0.0029$ and $P < 0.0001$ respectively) (Figure 1).

### Disease-free survival in advanced urothelial cancer patients according to serum MMP-2, TIMP-2 levels and MMP-2/TIMP-2 ratios

There was no significant difference in disease-free survival between the patients with elevated serum levels of MMP-2 and those with normal levels of the enzyme (Figure 2A). The serum level of TIMP-2 itself was also not correlated with disease-free survival (Figure 2B). Among the patients with advanced urothelial cancer, 19 patients had high MMP-2/TIMP-2 ratios (≥ 11.0) and 34 showed normal ratios (< 11.0). The 1- and 3-year disease-free survival rates of the patients with high MMP-2/TIMP-2 ratios were 50% and 12%, respectively, significantly unfavourable compared with those with a low ratio (82% and 56% respectively) ($P = 0.0152$) (Figure 2C).

### Univariate and multivariate analyses for recurrence in advanced urothelial cancer patients who underwent complete resection

The univariate analysis determined that significant predictors of recurrence were the serum MMP-2/TIMP-2 ratio ($P = 0.0228$), pT stage ($P = 0.0056$) and lymph node metastasis ($P = 0.0448$) (Table 3). The serum MMP-2/TIMP-2 ratio ($P = 0.0249$), pT stage

*© Cancer Research Campaign 1998*

**Figure 1** The serum MMP-2/TIMP-2 ratios in the 44 superficial bladder cancer and 53 advanced urothelial cancer patients. The mean serum MMP-2/TIMP-2 ratios in the advanced urothelial cancer patients with recurrence was significantly higher than that in the advanced urothelial cancer patients without recurrence and in the superficial bladder cancer patients ($P = 0.0029$ and ($P < 0.0001$, Mann–Whitney $U$-test). Data are means ± standard deviation

($P = 0.0011$), and lymph node metastasis ($P = 0.0260$) were also defined as significant independent predictors of recurrence using multivariate analysis.

### DISCUSSION

MMP-2 is one of the important proteases that degrades vascular basement membranes and extracellular matrices in the multiple metastatic process (Liotta et al, 1991; Nakajima et al, 1991; DeClerck et al, 1994). This enzyme also enhances the infiltration and migration of vascular endothelial cells, and thus induces neovascularization in malignant tumours (Liotta et al, 1991; Nakajima et al, 1991; DeClerck et al, 1994). The levels of MMP-2 and MMP-9 in high-grade or invasive bladder cancers were found to be significantly higher than those in low-grade and non-invasive bladder cancers, indicating that MMP-2 and MMP-9 may play a role in the invasion and metastasis of bladder cancer (Davies et al, 1993).

TIMPs are known for their anti-neoplastic activity (Albini et al, 1991; Montgomery et al, 1994). The transfection of sense TIMP-1 complementary DNA into metastatic cells decreased their invasive, tumorigenic and metastatic capacities (DeClerck et al, 1992; Khokha et al, 1994). Moreover, TIMPs are reported to inhibit angiogenesis, probably by affecting endothelial cell invasion, migration and proliferation (Moses et al, 1990; Takigawa et al, 1990; Liotta et al, 1991; DeClerck et al, 1994). The high level of

A

B

C

**Figure 2** Disease-free survival of the 53 advanced urothelial cancer patients after complete resection according to the serum levels of MMP-2 (**A**), TIMP-2 (**B**) and MMP-2/TIMP-2 ratios (**C**). The 1- and 3-year disease-free survival rates of the patients with high MMP-2/TIMP-2 ratios (≥ 11.0) were significantly unfavourable compared with the patients with lower ratios (< 11.0)($P = 0.0152$; log-rank test)

**Table 3** Prognostic factors of recurrence (univariate and multivariate analyses)

| | Disease-free survival | | | | | |
|---|---|---|---|---|---|---|
| | Univariate | | | Multivariate | | |
| | Hazard ratio | (95% CI) | P-value | Hazard ratio | (95% CI) | P-value |
| MMP-2/TIMP-2 (<11.0 vs ≥ 11.0) | 2.31 | (1.12–4.74) | 0.0228 | 2.39 | (1.12–5.10) | 0.0249 |
| Age (<61 years vs ≥ 61 years) | 1.14 | (0.54–2.43) | 0.734 | 2.18 | (0.91–5.22) | 0.0815 |
| Sex (Male vs female) | 0.91 | (0.37–2.22) | 0.835 | 0.998 | (0.37–2.68) | 0.999 |
| Grade (G1 vs G2 vs G3) | 0.93 | (0.46–1.85) | 0.826 | 0.73 | (0.32–1.65) | 0.446 |
| Stage (T1 vs T2 vs T3 vs T4) | 1.95 | (1.22–3.14) | 0.0056 | 2.94 | (1.54–5.61) | 0.0011 |
| Lymphovascular involvement (negative vs positive) | 1.58 | (0.75–3.31) | 0.231 | 0.74 | (0.28–2.01) | 0.5622 |
| Lymph node metastasis (negative vs positive) | 2.15 | (0.99–4.69) | 0.0448 | 2.78 | (1.13–6.84) | 0.0260 |

CI, confidence intervals.

TIMP-2 expression as detected by immunohistochemistry was associated with poor outcome in invasive bladder cancer patients (Grignon et al, 1996). However, several investigators have shown that the balance between MMPs and their inhibitors (TIMPs) modulated endothelial cell morphogenesis in vitro, and that TIMPs inhibited the early events in tube formation by endothelial cells on Matrigel (Schnaper et al, 1993). Therefore, MMPs and TIMPs in circulating body fluids may also contribute to the regulation of tumour metastasis, invasion and angiogesesis (Liotta et al, 1991; DeClerck et al, 1994).

The purpose of the present study was to determine whether the serum MMP-2/TIMP-2 ratio could be a predictor of invasion, metastasis and recurrence in advanced urothelial cancer patients who have undergone complete resection. Our results demonstrated that the serum levels of MMP-2 and TIMP-2 in the advanced urothelial cancer patients were significantly higher than those in the superficial bladder cancer patients. Moreover, the serum MMP-2/TIMP-2 ratio in the advanced urothelial cancer patients with recurrence was significantly higher than those in the superficial bladder cancer and advanced urothelial cancer patients without recurrence. In fact, the median disease-free survival of the patients with higher values of MMP-2/TIMP-2 (≥ 11.0) was significantly shorter than that of the patients with normal values (< 11.0). However, the disease-free survival was not correlated with the low level of serum MMP-2. In addition, although the results shown in Figure 2B suggest a possible correlation between the serum TIMP-2 level and the disease-free survival, high levels of serum TIMP-2 alone were not significantly correlated with the disease-free survival. Nevertheless, the imbalance of these enzymes and inhibitors is most likely an important factor in urothelial cancer invasion and metastasis, and, thereby, recurrence. There have been several studies on the inhibition of tumour invasion and metastasis by TIMP-1 (Schultz et al, 1988; Alvarez et al, 1990) and TIMP-2 (Albini et al, 1991; DeClerck et al, 1991). Koop et al (1994) found that the decreased metastatic ability of TIMP-overexpressing B16F10 melanoma cells was due to the effects of TIMP on tumour growth after tumour cell extravasation in the metastatic target organ. TIMP-2 has been shown to inhibit the basic fibroblast growth factor stimulation of endothelial cell growth (Murphy et al, 1993). In addition, synthetic MMP inhibitors have been demonstrated to suppress primary tumour growth (Naito et al, 1994; Wang et al, 1994). Therefore, MMP inhibitors could be inhibitory against not only tumour invasion but also tumour growth, through the inactivation of cell-associated MMPs.

In the present study, we noted that when the serum MMP-2/TIMP-2 ratio had been within the normal range (< 11.0), the secondary tumour at any metastatic site did not grow well. Therefore, even if micrometastatic lesions were formed before the operation, high levels of serum TIMP-2 would prevent metastatic tumour cells from developing further visible colonies.

Using multivariate analysis of recurrence we found that the serum MMP-2/TIMP-2 ratio is a new independent predictor comparable with traditional prognostic factors such as pT stage and lymph node metastatic status (Skinner et al, 1991).

In conclusion, our results indicate that the imbalance of serum MMP-2 and TIMP-2 (the MMP-2/TIMP-2 ratio) could be a new predictor of recurrence and may help us to determine whether or not patients with advanced urothelial cancer need intensive therapy, such as adjuvant chemotherapy, after complete resection.

### REFERENCES

Albini A, Melchiori A, Santi L, Liotta LA, Brown PD and Stetler-Stevenson WG (1991) Tumor cell invasion inhibited by TIMP-2. *J Natl Cancer Inst* **83**: 775–779

Alvarez OA, Carmichael DF and DeClerck YA (1990) Inhibition of collagenolytic activity and metastasis of tumor cells by a recombinant human tissue inhibitor of metalloproteinase. *J Natl Cancer Inst* **82**: 589–595

Cox DR (1972) Regression models and life table. *J Roy Stat Soc (B)* **34**: 187–220

Davies B, Waxman J, Wasan H, Abel P, Williams G, Krausz T, Neal D, Thomas D, Hanby A and Balkwill F (1993) Levels of matrix metalloproteinases in bladder cancer correlate with tumor grade and invasion. *Cancer Res* **53**: 5365–5369

DeClerck YA and Imren S (1994) Protease inhibitors: role and potential therapeutic use in human cancer. *Eur J Cancer* **30A**: 2170–2180

DeClerck YA, Yean TD, Chan D, Shimada H and Langley KE (1991) Inhibition of tumor invasion of smooth muscle cell layers by recombinant human metalloproteinase inhibitor. *Cancer Res* **51**: 2151–2157

DeClerck YA, Perez N, Shimada H, Boone TC, Langley KE and Taylor SM (1992) Inhibition of invasion and metastasis in cells transfected with an inhibitor of metalloproteinases. *Cancer Res* **52**: 701–708

Fujimoto N, Mouri N, Iwata K, Ohuchi E, Okada Y and Hayakawa T (1993*a*) A one-step sandwich enzyme immunoassay for human matrix metalloproteinase 2 (72-kDa gelatinase/type IV collagenase) using monoclonal antibodies. *Clin Chim Acta* **221**: 91–103

Fujimoto N, Zhang J, Iwata K, Shinya T, Okada Y and Hayakawa T (1993*b*) A one-step sandwich enzyme immunoassay for tissue inhibitor of metalloproteinases-2 using monoclonal antibodies. *Clin Chim Acta* **220**: 31–45

Grignon DJ, Sakr W, Toth M, Ravery V, Angulo J, Shamsa F, Pontes JE, Crissman JC and Fridman R (1996) High levels of tissue inhibitor of metalloproteinase-2 (TIMP-2) expression are associated with poor outcome in invasive bladder cancer. *Cancer Res* **56**: 1654–1659

Khokha R (1994) Suppression of the tumorigenic and metastatic abilities of murine B16-F10 melanoma cells in vivo by the overexpression of the tissue inhibitor of the metalloproteinases-1. *J Natl Cancer Inst* **86**: 299–304

© Cancer Research Campaign 1998

Koop S, Khokha R, Schmidt EE, MacDonald IC, Morris VL, Chambers AF and Groom AC (1994) Overexpression of metalloproteinase inhibitor in B16F10 cells does not affect extravasation but reduces tumor growth. *Cancer Res* **54**: 4791–4797

Liotta LA, Tryggvason K, Garbisa S, Hart I, Foltz CM and Shafie S (1980) Metastatic potential correlates with enzymatic degradation of basement membrane collagen. *Nature* **284**: 67–68

Liotta LA, Steeg PS and Stetler-Stevenson WG (1991) Cancer metastasis and angiogenesis: an imbalance of positive and negative regulation. *Cell* **64**: 327–336

Montgomery AMP, Muller BM, Reisfeld RA, Taylor SM and DeClerck YA (1994) Effect of tissue inhibitor of the matrix metalloproteinases-2 expression on the growth and spontaneous metastasis of a human melanoma cell line. *Cancer Res* **54**: 5467–5473

Moses MA, Sudhalter J and Langer R (1990) Identification of an inhibitor of neovascularization from cartilage. *Science* **248**: 1408–1410

Murphy AN, Unsworth EJ and Stetler-Stevenson WG (1993) Tissue inhibitor of metalloproteinases-2 inhibits bFGF-induced human microvascular endothelial cell proliferation. *J Cell Physiol* **157**: 351–358

Naito K, Kanbayashi N, Nakajima S, Murai T, Arakawa K, Nishimura S and Okuyama A (1994) Inhibition of growth of human tumor cells in nude mice by a metalloproteinase inhibitor. *Int J Cancer* **58**: 730–735

Nakajima M and Chop AM (1991) Tumor invasion and extracellular matrix degradative enzymes; regulation of activity by organ factors. *Semin Cancer Biol* **2**: 115–127

Schnaper HW, Grant DS, Stetler-Stevenson WG, Fridman RD, Orazi G, Murphy AN, Bird RE, Hoythya M, Fuerst TR, French DL, Quigley JP and Kleinman HK (1993) Type IV collagenase(s) and TIMPs modulate endothelial cell morphogenesis in vitro. *J Cell Physiol* **156**: 235–246

Schultz RM, Silberman S, Persky B, Bajkowski AS and Carmichael DF (1988) Inhibition by human recombinant tissue inhibitor of metalloproteinases of human amnion invasion and lung colonization by murine B16-F10 melanoma cells. *Cancer Res* **48**: 5539–5545

Skinner DG, Daniels JR, Russell CA, Lieskovsky G, Boyd SD, Nichols P, Kern W, Sakamoto J, Krailo M and Groshen S (1991) The role of adjuvant chemotherapy following cystectomy for invasive bladder cancer: a prospective comparative trial. *J Urol* **145**: 459–467

Stetler-Stevenson WG, Krutzsch HC and Liotta LA (1989) Tissue inhibitor of metalloproteinase (TIMP-2). A new member of the metalloproteinase family. *J Biol Chem* **264**: 17374–17378

Takigawa M, Nishida Y, Suzuki F, Kishi J, Yamashita K and Hayakawa T (1990) Induction of angiogenesis in chick yolk-sac membrane by polyamines and its inhibition by tissue inhibitors of metalloproteinases (TIMP and TIMP-2). *Biochem Biophys Res Commun* **171**: 1264–1271

Wang X, Fu X, Brown PD, Crimmin MJ and Hoffman RM (1994) Matrix metalloproteinase inhibitor BB-94 (Batimastat) inhibits human colon tumor growth and spread in a patient-like orthotopic model in nude mice. *Cancer Res* **54**: 4726–4728

© Cancer Research Campaign 1998